# The Influence of Toothpaste Containing Australian *Melaleuca alternifolia* Oil and Ethanolic Extract of Polish Propolis on Oral Hygiene and Microbiome in Patients Requiring Conservative Procedures

**DOI:** 10.3390/molecules22111957

**Published:** 2017-11-13

**Authors:** Tomasz Piekarz, Anna Mertas, Karolina Wiatrak, Rafał Rój, Patryk Kownacki, Joanna Śmieszek-Wilczewska, Ewelina Kopczyńska, Maciej Wrzoł, Maria Cisowska, Ewelina Szliszka, Zenon P. Czuba, Iwona Niedzielska, Tadeusz Morawiec

**Affiliations:** 1 School of Medicine with the Division of Dentistry in Zabrze, Medical University of Silesia in Katowice, Pl. Traugutta 2, 41-800 Zabrze, Poland; tomasz_lek_dent@interia.pl (T.P.); szczepa.karol@gmail.com (K.W.); ewelina.kopczynska90@gmail.com (E.K.); 2 Department of Microbiology and Immunology, School of Medicine with the Division of Dentistry in Zabrze, Medical University of Silesia in Katowice, Jordana 19, 41-808 Zabrze, Poland; m.cisowska4@wp.pl (M.C.); eszliszka@sum.edu.pl (E.S.); zczuba@sum.edu.pl (Z.P.C.); 3 Department of Prosthetic Dentistry, School of Medicine with the Division of Dentistry in Zabrze, Medical University of Silesia in Katowice, Plac Akademicki 17, 41-902 Bytom, Poland; rafstoma@gmail.com; 4 Department of Oral Surgery, School of Medicine with the Division of Dentistry in Zabrze, Medical University of Silesia in Katowice, Pl. Akademicki 17, 41-902 Bytom, Poland; pkownacki@sum.edu.pl (P.K.); tallen@poczta.onet.pl (J.Ś-W.); mwrzol@sum.edu.pl (M.W.); tmorawiec@sum.edu.pl (T.M.); 5 Department and Hospital of Craniomaxillofacial Surgery and Oral Surgery, School of Medicine with the Division of Dentistry in Zabrze, Medical University of Silesia in Katowice, Pl. Akademicki 17, 41-902 Bytom, Poland; niedzielska.konsultant@wp.pl

**Keywords:** tea tree oil, propolis, oral hygiene, oral microbiome

## Abstract

The study was based on the use of a toothpaste with antiphlogistic activity, containing Australian *Melaleuca alternifolia* oil (tea tree oil—TTO) and ethanolic extract of Polish propolis (EEP). Fifty-one patients with varying conditions of the gingiva were divided into two groups. The study group received the toothpaste with TTO and EEP, while the control group received the same toothpaste but without TTO and EEP. Approximal plaque index (API), simplified oral hygiene index (OHI-s) and modified sulcus bleeding index (mSBI) were assessed in three subsequent stages. During each examination, swabs were employed for microbiological inoculation. During the period of use of toothpastes with TTO and EEP, a significant reduction of the API was observed, as assessed upon the control visit after 7 days and after 28 days, compared to baseline. A statistically significant reduction of mSBI was observed after 7 and 28 days of using the toothpaste with TTO and EEP, as compared to the value upon the initial visit. Statistically significant differences in the OHI-s value were observed in the study group, which was using the active toothpaste. The use of a toothpaste containing TTO and EEP helps to maintain microbiome balance. The observed stabilisation of bacterial microflora confirms the beneficial activity of toothpaste containing EEP and TTO compared to the control group, where the lack of these substances contributed to the emergence of qualitative and quantitative changes in oral microbiome.

## 1. Introduction

Homeostatic disruptions in the oral cavity may occur as a result of quantitative or qualitative imbalance of endogenous microflora or due to the transfer of exogenous microflora. There are over 700 species of microorganisms dwelling in the oral cavity, which pose no health threat in physiological balance. The normal microflora of the oral cavity includes the following genera of bacteria: *Streptococcus*, *Granulicatella*, *Gemella*, *Veillonella*, *Leptotrichia*, *Neisseria*, *Campylobacter*, *Porphyromonas*, *Prevotella*, *Fusobacterium*, *Eubacterium*, *Actinomyces*, *Rothia*, *Simonsiella*, *Bacteroides*, *Peptostreptococcus*, *Capnocytophaga* [1,2,3]. Oral hygiene has a major influence on general health and the quality of life. There are proven correlations between oral health and systemic diseases, which concern inter alia, circulatory, respiratory, endocrine and musculoskeletal systems [4,5]. Pathogenic microorganisms, their toxins and inflammatory mediators may penetrate from the focus of infection into the bloodstream, influencing general health, which is supported by the focal infection theory [6]. Therefore, proper oral hygiene and following the rules of aseptics and antiseptics is of great importance in dentistry. Prevention, education on proper oral hygiene and raising patients’ awareness of the consequences of neglecting it are among the most important stages in conservative treatment. Maintenance of optimal oral hygiene consists of the following procedures: flossing of interdental spaces, proper tooth brushing and rinsing of the oral cavity. Negligence of oral hygiene leads to diseases of the gingiva and periodontium as well as development of caries and consequently progression of untreated carious cavities, which in turn leads to pulpitis and endodontic therapy. Thus, prophylaxis and treatment of early carious lesions are extremely important [7,8]. Brushing technique is also significant for proper mechanical removal of plaque. The removal is assisted by chemicals contained in toothpastes and rinses [9]. There are numerous toothpastes and rinses with different compositions on the market. Toothpastes contain, e.g., fluorine, triclosan and xylitol, whereas rinses contain chlorhexidine, cetylpyridinium, alcohol, essential oils and herbal components [10,11,12,13,14,15,16]. Natural ingredients are gaining in popularity as components of toothpastes and rinses. Many studies confirm the antimicrobial and antiphlogistic activity of preparations containing propolis and tea tree oil (TTO), which have a positive impact on oral hygiene as toothpastes or rinses [17,18,19]. Propolis is a resinous substance produced by honey bees (*Apis mellifera*) with antimicrobial, antiviral, antifungal and antiphlogistic properties [20]. It contains, inter alia: phenols, esters, aromatic alcohols, fatty acids, β-steroids, mineral salts and vitamins. Propolis from different geographical zones differs in its chemical composition and biological activity mainly due to varying plant materials from which it is produced [21]. Thanks to its properties, it has a wide range of applications in medicine, including dentistry. It is used, e.g., in periodontal diseases, to prevent from caries, reduce mucositis in patients subjected to chemotherapy, reduce dentin hypersensitivity and is a component of dentifrices [22]. TTO is obtained from leaves of the *Melaleuca alternifolia* plant. Two other species (*Melaleuca linariifolia* and *Melaleuca dissitiflora*) are also used to obtain the essential oil, only if they contain more than 30% terpinen-4-ol in the essential oil from the leaves. The main active ingredient of TTO is terpinen-4-ol. Its content is between 29–45%. Structurally similar compounds occur in smaller amounts: γ-terpinene (12–23%), α-terpinene (8–11%), α-terpineol (2–7%) and monoterpenes, such as 1,8-cineole (2–16%), *p*-cymene (1–12%), α-pinene (2–5%) and limonene (1–6%). 1,8-cineole is considered to be irritating to the skin, while terpinen-4-ol and α-terpineol are strong antimicrobial compounds. Chemical composition of TTO used for production of medicinal products has to meet the ISO 4730:2004 requirements [23]. TTO has many useful properties. It has an analgesic effect, accelerates wound healing and exhibits antimicrobial activity, particularly antifungal [24,25].

The aim of this study was to evaluate the influence of toothpaste with active substances of plant origin, ethanolic extract of propolis and tea tree oil, on the microbiome and to calculate the values of selected oral indices (simplified Oral Hygiene Index—OHI-s, Approximal Plaque Index—API, Sulcus Bleeding Index—mSBI) in patients treated with use of conservative and preventive procedures.

## 2. Results

**Figure 1:**
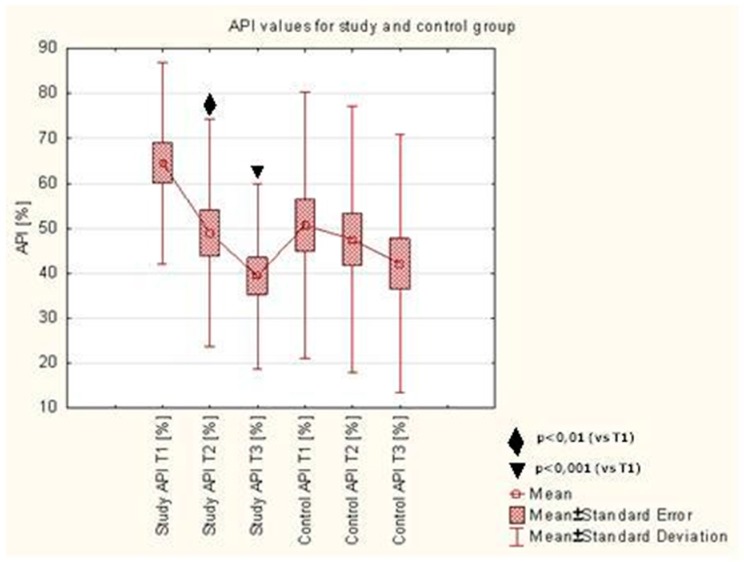
Approximal plaque index (API) values in study and control groups.

API assessed upon initial visit (T1) in the study group was 64.58 ± 22.38%. During the use of toothpastes with ethanolic extract of propolis (EEP) and TTO (AT preparation), a significant reduction of API was observed, as assessed upon the control visit after 7 days of using the preparation (49.00 ± 25.32%, *p* < 0.006) and after 28 days (39.39 ± 20.60%, *p* < 0.0002), compared to baseline. No statistically significant reduction of the index assessed after 7 (T2) and 28 (T3) days was observed in the control group. There were no differences observed between the API values after 7 and 28 days of using the AT preparation in the study group or the CT preparation in the control group. Detailed results were presented in Table 1 and Figure 1.

**Figure 2:**
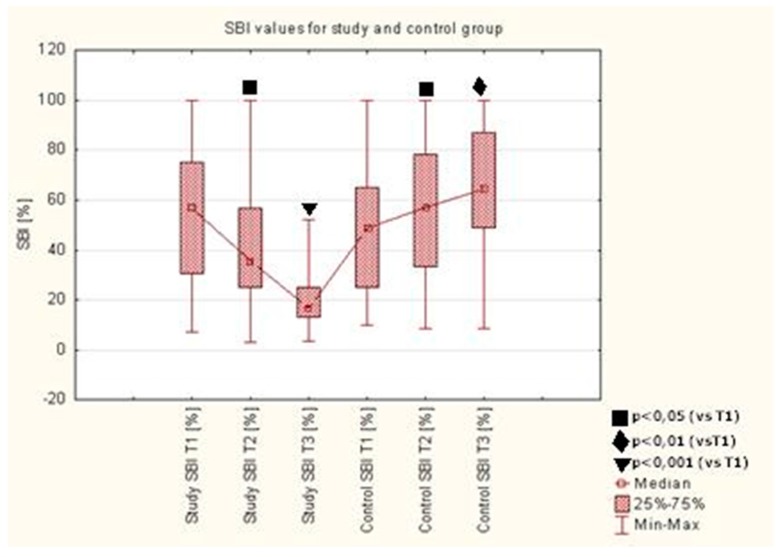
Modified sulcus bleeding index (mSBI) values in study and control groups.

A statistically significant reduction of mSBI was observed after 7 (T2) and 28 (T3) days of using the toothpaste with EEP and TTO (AT preparation) in the study group, as compared to the value upon the initial visit (T1) (*p* < 0.05, *p* < 0.0001). Detailed results are presented in Table 2 and Figure 2.

**Figure 3:**
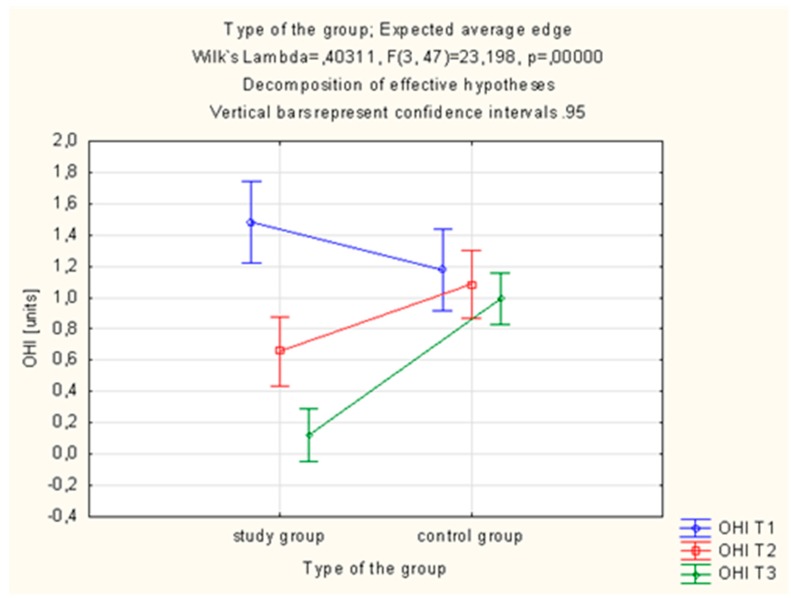
ANOVA Estimated Marginal Means for the simplified oral hygiene index (OHI-s).

Statistically significant differences in the OHI-s value were observed in the study group, which was using the active toothpaste (AT preparation). In this group, OHI-s was statistically significantly lower after 28 days (T3) of using the AT toothpaste (1.48 ± 0.70 vs. 0.12 ± 0.18%; *p* = 0.000001, (Table 3, Figure 3).

One-way analysis of variance ANOVA was employed in order to verify whether the value of indices differs statistically significantly in particular periods of the study after the use of toothpaste with ethanolic extract of propolis (EEP) and tea tree oil (TTO). It demonstrated a statistically significant difference in OHI-s between the 7th and 28th day of the study. Homogeneity of variance was obtained in the 28th day of the study. Detailed data is presented in Table 4.

**Figure 4:**
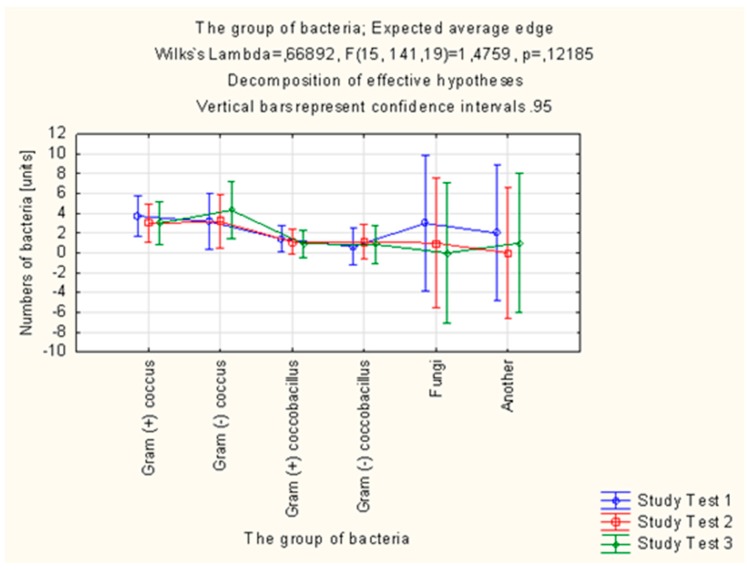
Distribution of isolated microorganisms in the study group.

**Figure 5:**
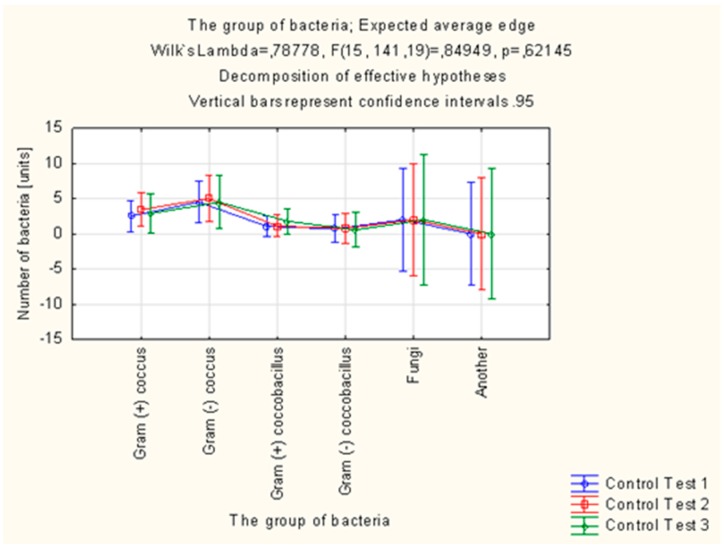
Distribution of isolated microorganisms in the control group.

Altogether, 305 strains of bacteria were isolated in the study group in the microbiological examinations of smears from mouth floor mucosa. Their number was decreasing in each subsequent examination. In total, 321 strains of bacteria were isolated in the control group, the number of which was increasing in each subsequent examination. *Candida albicans* fungi were successfully eradicated in the group of patients using the active preparation (AT), as opposed to the control group. The number of *Streptococcus mitis* isolates decreased in the study group and increased by 43.75% in the control group between the first (T1) and third (T3) examination. In the case of *Streptococcus sanguinis*, the number of isolates in the final examination (T3) was higher than in the initial examination (T1) in the control group, while the number of isolates of this species decreased in the study group. *Streptococcus sanguinis* and *Streptococcus mitis* are considered to be pioneer microorganisms, responsible for the development of plaque. The number of *Actinomyces israelii* and *Actinomyces naeslundii* isolates in the control group was significantly higher in the final examination (T3) than in the initial examination (T1). The increase in the number of these microorganisms promotes the progressive development of plaque. Bacteria of the *Campylobacter gracilis* species, responsible, e.g., for the development of gingivitis, were eradicated in the study group, while the number of isolates of this species increased in the control group (Table 5, Figure 4 and Figure 5).

## 3. Discussion

Modern medicine and dentistry employ synthetic drugs, which have dominated the world of medicine. However, natural ingredients have been gaining in popularity over recent years. Studies are conducted, which show how valuable and efficient natural products are. An increasing number of research centres concentrate their studies on such products [26,27,28]. Naturally occurring substances are also increasingly common in treating various dental diseases. The application of ethanolic extract of propolis (EEP) and tea tree oil (TTO) in treating diseases of the oral cavity, skin and mucous membranes has been the subject of numerous studies. EEP has many useful properties, e.g., antimicrobial, antiviral, antifungal, antiphlogistic and antioxidant activity. It exhibits antibacterial activity against cariogenic bacteria: *Streptococcus mutans* and *Lactobacillus* spp. [29], and thus is frequently and successfully used as an active ingredient of toothpastes and mouthwashes [30,31]. Capistrano et al. conducted a study showing the effect of propolis on *Candida albicans* fungi. It demonstrated that propolis has a similar efficiency to miconazole in treating tinea in patients with dentures [32]. Carbajal Mejía proved that propolis, similarly to chlorhexidine, acts against *Enterococcus faecalis*, a bacterium responsible for numerous failures in endodontic treatment [33]. There are also reports claiming that propolis reduces oral mucositis in patients undergoing radiotherapy. Research conducted by Javadzadeh Balouri et al. confirmed that propolis prevents and is efficient in treating radiotherapy-induced mucositis [34]. Another significant activity of propolis is its antiviral effect. Yildirim et al. demonstrated the inhibitory effect of propolis on proliferation of HSV1 and HSV2 (HSV—Herpes Simplex Virus). Its efficiency is similar to that of acyclovir and combination of propolis and acyclovir increases the antiviral effect [35]. A naturally occurring substance with multidirectional biological activity is also tea tree oil (TTO), obtained from two genera of trees of the *Myrtaceae* family: *Leptospermum* and *Melaleuca* [36]. Tea tree oil has antimicrobial and antiphlogistic properties, as well as inhibiting the development of *Candida albicans*. Ramage et al. confirmed the antifungal effect of TTO in their research [37]. Pereira et al. proved that tea tree oil is efficient against human pathogens [38].

In studies conducted for the purpose of this article, a significant reduction of the API was observed during the use of toothpastes with EEP and TTO, as assessed upon the control visit after 7 days of using the preparation and after 28 days, compared to baseline. No statistically significant reduction of the index assessed after 7 and 28 days was observed in the control group. API determines the presence or absence of a bacterial plaque in interdental spaces. During the first visit, patients received the control or test toothpaste and were educated on oral hygiene. Both toothpastes contain abrasives while the control paste has no active ingredients. Patients who properly clean their teeth, with either a test or control paste, can achieve a comparable mechanical cleaning effect. In their study, Niedzielska et al. [39] used a gel with 3% ethanolic extract of Brazilian green propolis in patients who had undergone osteoinductive treatment of mandible fractures. Authors were investigating the changes in API on postoperative days 1, 8 and 22. They noticed a statistically significant decrease in API between the first and second, second and third, as well as first and third examination. Authors of the article reported larger fluctuations of this index in the control group compared to the results of this study. The decrease in the API value on a statistically significant level could have been associated with greater care paid to oral hygiene by patients after surgical procedures than those after conservative procedures. The study by Skaba et al. [19] conducted on a group of patients with use of a toothpaste with 3% ethanolic extract of propolis showed an improvement in API, although without marked statistical significance. Morawiec et al. [40] conducted studies with use of a toothpaste with 3% ethanolic extract of propolis in patients after implant rehabilitation. The assessment of oral hygiene indices was performed after 1 and 8 weeks from the beginning of the study. A decreasing trend in the API value was observed in both control and study groups. Statistical correlation was observed only in the results of the study group between the first and last measurements. On the first visit, the mean oral hygiene in patients of the study group was classified as “average oral hygiene”, whereas after the study the mean API value indicated the “optimal hygiene” condition. The vast majority of patients in the control group had “average oral hygiene” and “quite good oral hygiene”. Machorowska-Pieniążek et al. [18] carried out a study with use of a 3% toothpaste with ethanolic extract of propolis in patients treated orthodontically. They measured the API in the first and 35th day of the study. Statistical significance of the positive changes in the value of this index in study and control groups was not observed. Improvement in the API could have been associated with the low average age of the patients (12.4 years old), since younger patients with orthodontic restorations can have trouble maintaining proper oral hygiene. A study of oral health was conducted by Tanasiewicz et al. [41] with use of 3% ethanolic extract of propolis contained in a gel. API values (according to Lange), in the case of patients who were to begin using an active and control gel, were within the limits of positive values on the first visit. Patients in the group with the active preparation were classified within the range described as “quite good hygiene”, while during the observation time (visit 7 days after the first examination) their results changed to the range described as “optimal hygiene”. Moreover, on the third visit, the results went back to the range of “quite good hygiene”. Group II (the placebo group) patients were classified as “average hygiene” on the first examination. It remained unchanged during all subsequent examinations. In the study conducted for the purpose of this article, 12% of the patients in the study group were classified as “quite good hygiene”. This percentage rose to 52% of study subjects on the third examination. In total, 19% of patients in the control group were described as having “quite good hygiene”, which fell to 12% upon the third examination.

A statistically significant reduction of mSBI was observed after 7 and 28 days of using the toothpaste with EEP and TTO in the study group, as compared to the value upon the initial visit (T1). The mSBI index defines gum inflammation and gingival bleeding. The study group was using an active paste containing therapeutic ingredients. These ingredients caused the reduction of gum inflammation and gingival bleeding so the mSBI index dropped. The control group was using a neutral paste and the mechanical brushing only did not reduce the inflammation. Patients in the control group, while using the inactive toothpaste, did not inhibit the gingival inflammation, so the gingival bleeding and consequently mSBI index increased. Similar statistically significant changes in SBI were observed by other authors [39] as a decrease in SBI on postoperative days 8 and 22 compared to the first day and between days 8 and 22. Also, Skaba et al. [19] observed a non-statistically significant decrease in SBI mean values. The improvement between the first and second examination did not maintain the trend between the second and third examination. These changes in comparison with the studies conducted for this article have the same index value change vector, while the decrease of value over time is less significant. This might be caused by the difference in the number of patients in the groups examined or by worse discipline among the patients. The study by Morawiec et al. [40] did not demonstrate a statistically significant improvement in the SBI either in the study or in the control group. The discrepancy between that observation and the results of this study might be a result of the original group selection. In the study by Morawiec et al. [40], 100% of the control group were classified as “healthy gingiva, no bleeding on probing” with the SBI value <10%. In our study, both study and control groups included 96% of patients classified as “bleeding on probing” with SBI >10%. A study of oral health conducted by Tanasiewicz et al. [41] with use of 3% ethanolic extract of propolis in the toothpaste demonstrated a change in the SBI after 1 and 8 weeks of the study. Patients in the group using the active preparation and placebo were within the ranges “healthy gingiva, no bleeding on probing” and “bleeding on probing, no changes in colour and shape” upon the first examination. All patients were described as “healthy gingiva, no bleeding on probing” upon the third examination. However, this trend did not demonstrate statistical correlation. In our study, the percentage of patients with “healthy gingiva” rose from 4% to 24% within the group using the active preparation with EEP and TTO. The increase in the control group was from 4% to 8%.

Statistically significant differences in the OHI-s value were also observed in the study group, which was using the active toothpaste. In this group, OHI-s proved to be statistically significantly lower after 28 days of using the active toothpaste. Statistically significant differences were obtained by Niedzielska et al. [39], who obtained a statistically significant decrease in OHI-s in the control group. Discrepancies in observations between the above-mentioned studies and our study might be associated with specific study groups. The hygiene regimen is more comprehensive and strictly adhered to in surgical patients than in patients undergoing conservative treatment. Studies conducted by Skaba et al. [19] with use of 3% toothpaste containing ethanolic extract of Brazilian propolis demonstrated a decrease in the OHI-s value in weeks 1 and 4 of using the active preparation. The observed decrease was statistically significant between the first and third, first and second as well as second and third examination, In the study by Morawiec et al. [40], with use of a toothpaste with 3% ethanolic extract of Brazilian green propolis in patients after implant-prosthetic rehabilitation, an improvement of OHI-s was observed in both examined groups. The authors did not notice a statistical significance of the changes observed. The authors point out the positive influence of both propolis itself contained in the preparation and correct hygienic behaviour arising from the hygienic training at the beginning of the study. The study by Tanasiewicz et al. [41] with use of 3% ethanolic extract of propolis contained in toothpaste and gel shows a significant improvement in the OHI-s value in the examined patients. Improvements both in control and study groups with use of the toothpaste with EEP were statistically significant. The authors suggest that such a result might be caused by patients without diagnosed periodontitis, only exhibiting tendencies to neglect oral hygiene, being encouraged by the participation in the study. Similar observations result from this study. The influence of patients’ will to maintain hygiene due to the participation in the study significantly improves hygiene and thus values of the indices. We obtained statistical correlation in the change of OHI-s, which might be related to worse values of this index at the early stage of the study than in the quoted article.

In our study, the API values as shown in Figure 1, and the OHI-s values as shown in Figure 3, decrease on 7 days and 28 days after initiation. API and OHI-s are indicators of oral hygiene. They depend on the presence or absence of plaque. API determines the presence or absence of a bacterial plaque in the interdental spaces and OHI-s shows the presence or absence of plaque and tartar on the smooth surfaces. Decreasing value of these indicators was associated with better oral hygiene. Patients who performed the oral hygiene instruction were better at brushing their teeth and that caused the reduction of plaque. What is more, patients knew that they would be examined during the visit and for this reason they brushed their teeth better before the visit. The composition of the preparation in both the control and the test group allowed for similar mechanical cleaning of the tooth surfaces and removal of the plaque. For this reason, API and OHI-s decreased after 7 days and 28 days in both groups.

In our study, the collection of material from patients’ mouth floor mucosa, for microbiological examination, was performed with a sterile swab equipped with a transport medium upon the initial visit (T1), after 7 days (T2) and after 4 weeks (T3) from the initial visit. Results of microbiological examinations conducted by Niedzielska et al. [39] demonstrated significant qualitative and quantitative differences in oral microflora of patients who used the 3% EEP gel for everyday oral care, as compared to patients using the gel without EEP. Similar changes in the composition of oral microflora after the use of the toothpaste with 1.5% EEP combined with 1% TTO were observed in our own study. Skaba et al. [19] conducted an in vitro study intended to assess the activity of Brazilian EEP against the following bacteria: *Streptococcus mutans*, *Staphylococcus aureus*, *Lactobacillus acidophilus*, *Aggregatibacter actinomycetemcomitans*, *Streptococcus sanguinis*, *Porphyromonas gingivalis* and fungi: *Candida albicans*. A significant antimicrobial activity was observed, which was increasing over time as the EEP was active. The study by Machorowska-Pieniążek et al. [18] with the use of a toothpaste with 3% ethanolic extract of Brazilian propolis conducted in patients undergoing orthodontic treatment demonstrated that the most frequently isolated bacteria from the oral cavity in both groups and at both stages of the study were *Streprococcus* spp. and *Neisseria* spp. The presence of *Actinomyces* spp. together with *Actinomyces israelii*, *Capnocytophaga* spp., *Fusobacterium* spp., *Bacteroides* spp. and *Eubacterium* spp. was observed among bacteria pathogenic for the periodontium. A statistically lower number of *Actinomyces* spp. and *Capnocytophaga* spp. isolates was observed in patients using the toothpaste with EEP at the second stage of the study. The number of *Candida albicans* fungi isolates remained the same. In our own study, a microflora of similar composition was isolated from the study subjects, while the number of *Actinomyces* spp. isolates in the study group was without significant changes during the study. An increase in the number of isolates in subsequent examinations was observed in the control group. The observed stabilisation of bacterial microflora confirms the beneficial activity of toothpaste containing EEP and TTO compared to the control group, where the lack of these substances contributed to the emergence of qualitative and quantitative changes in oral microbiome. In our own study, there was a decrease in the number of *Candida albicans* isolates in patients using both the active toothpaste and the placebo.

Caries is a disease of extracorporeal origin that leads to decalcification and proteolytic breakdown of hard tooth tissue. The factors that cause caries development and progress include time, substrate (sugars), bacteria, and susceptibility of hard tissues to caries. The hard tissues of the tooth are destroyed by the organic acids that are produced by the bacteria. Caries are formed when demineralisation processes outweigh remineralisation. Bacterial plaque is formed shortly after proper cleaning of the teeth. In the first stage of the formation of the bacterial plaque, which is carried out under aerobic conditions, Gram positive grains are involved. These include *Streptococcus oralis*, *Streptococcus mitis*, *Streptococcus sanguinis*, *Streptococcus gordonii* and *Streptococcus mutans*. Streptococci produce suitable receptors to attach themselves to other microorganisms, including *Actinomyces naeslundii*, *Eikenella corrodens*, and *Veillonella atypia.* In the later stages of the formation of the bacterial plaque, the oxidative potential is reduced and anaerobic conditions are produced. As a consequence, anaerobes and microaerophiles are prevalent in the plaque, including *Veillonella atypica*, *Fusobacterium nucleatum*, *Corynebacterium* sp., *Leptotrichia* sp., *Prevotella* sp., *Porphyromonas* sp., *Treponema* sp. and *Aggregatibacter* sp. Mostly lactic acid and acetic acid are responsible for demineralization, that is, the loss of minerals from the enamel. They diffuse into the pores of the enamel and then dissociate, dissolving the superficial layers of enamel, interrupting their continuity, which is associated with the onset of caries formation. Wassel et al. [42] tested specially prepared dental varnishes containing propolis, miswak, and chitosan nanoparticles with or without sodium fluoride (NaF). The varnishes have been evaluated for their antibacterial activity against *Streptococcus mutans*. All varnishes containing natural products were reducing the growth of *Streptococcus mutans* bacteria. The authors concluded that the addition of natural products with fluoride to dental varnishes, especially miswak and propolis, can be an effective way to prevent caries. De Luca et al. [43] developed a propolis varnish, considering propolis properties against cariogenic bacteria. To a chitosan polymeric base was added ethanolic propolis extract in different concentrations (5%, 10% or 15%). Antimicrobial activity was carried out against *Streptococcus mutans*, *Streptococcus sanguinis*, *Streptococcus salivarius*, and *Lactobacillus casei.* Three concentrations of propolis were effective in inhibiting the growth of all microorganisms but without significant difference between the zones of inhibition. The varnishes had satisfactory antimicrobial activity against cariogenic bacteria and they have a low cytotoxicity (<50%). In our studies, we observed a similar effect of propolis formulation on microbiological status. We noted the reduction of *Streptococcus salivarius*, *Streptococcus sanguinis* and *Streptococcus oralis* between T1 and T3 examination in patients receiving the active formulation with propolis. These are cariogenic bacteria and the decrease in their amount reduces the susceptibility of hard tooth tissues to caries. Mohsin et al. [29] evaluated the antibacterial efficacy of propolis-based dentifrice on Streptococci Mutans infections, colonizing the oral cavity of young patients. Screening was performed in 367 men in the 7–12 age group. It was found that the mean number of *Streptococcus mutans* in 1 week and 4 weeks showed a significant decrease (*p* ≤ 00001), as compared to the results obtained in the control study. The propolis-containing dentifrice cleaner reduces the microbial load in vivo, especially against *Streptococcus mutans* in the oral cavity of young patients. In this way, propolis can be used as an alternative means of preventing dental caries.

Natural antimicrobial substances are currently gaining in popularity. Thanks to their properties, they are increasingly frequently employed and recommended to patients who report with various ailments and diseases, not only within the oral cavity. Products containing biologically active natural substances can be a very valuable complement used both in preventive and therapeutic measures.

## 4. Materials and Methods

### 4.1. Toothpaste Preparations

Two types of toothpaste (active and control), not available commercially, were produced specifically for the study. Toothpastes were in identical unmarked containers. The active toothpaste (AT) had the following qualitative composition: ethanolic extract of propolis (EEP) (1.5%), tea tree oil (TTO) (1.0%), menthol oil (0.2%) and rosemary oil (0.1%), glycerine (5–12%), silica (10–14%), sorbitol (10–20%), hydroxyethyl cellulose (0.1–1%), titanium dioxide (0.5–2%), xanthan gum (0.3–1%), aqua (up to 100% of weight). The control toothpaste (CT), a *placebo* preparation, did not contain any active substances, only ingredients of the toothpaste base (glycerine, silica, sorbitol, hydroxyethyl cellulose, titanium dioxide, xanthan gum, aqua). The following companies were responsible for developing the composition and production technology of the toothpastes: AS Cosmetics Sernice (Warsaw, Poland) and Melaleuca Poland Sp. z o.o. (Gliwice, Poland). Tea tree oil of Australian origin fulfilling the ISO 4730:2004 requirements was used in production of the active toothpaste, together with Polish propolis from apiaries located in north-western Poland, produced by *Apis mellifera* bees, fulfilling the Polish Standard for Propolis Concentrate PN-A-77627.

### 4.2. Patients

Clinical examinations were carried out between July 2015 and March 2016 in Private Dental Clinic ComfortMed (Katowice, Poland), responsible for comprehensive dental care of examined patients. Selection of research subjects was based on a single-blind trial, where the investigator was aware of the type of preparation administered to the patient. All materials used complied with the standards for approval for medical use. The study was conducted with prior approval from the Bioethics Committee of the Silesian Medical Chamber in Katowice, Poland, Resolution No. August 2015 of 23 March 2015.

The first stage of the study was to ascribe patients to proper study groups on the basis of a general medical interview, complemented by a dental interview and extraoral and intraoral examinations. The study was extended by a survey concerning medical history and current diseases, allergies, taken medication and environmental risk. Persons qualified for study or control groups were evaluated to determine the value of oral hygiene indices (OHI-s, API, mSBI). The procedure of determining the indices was non-invasive and entirely safe for the patients. It was performed with use of a standard WHO periodontal probe with a non-aggressive tip. Fifty-one patients were qualified for the research. They were adults of both sexes (33 women, 18 men) aged 18 to 75. Patients were generally healthy, treated in terms of conservative dentistry and endodontics, undergoing preventive procedures and non-invasive periodontal therapy of inflammatory lesions of the gingiva and periodontium. Exclusion criteria from the study were as follows: presence of chronic systemic diseases, cancer, psychosomatic disorders, patients after trauma within the craniofacial region, pregnant and lactating females, asthma, atopic dermatitis, allergies to foods, drugs, honey and bee products.

Study subjects were randomly divided into two groups. The study group of 25 received the active toothpaste (AT preparation, containing EEP and TTO). The control group of 26 received the control toothpaste (CT preparation—*placebo* preparation without active substances) in an identical container. Both groups were subjected to hygienisation of the oral cavity with use of supragingival scaling and instructed on how to properly brush teeth with use of the Fones circular technique, consisting of putting the toothbrush perpendicular to the gingiva and performing circular movements. This technique let the patients remove the biofilm from hard tooth tissue to the optimum extent. Patients were instructed to brush their teeth twice a day with the received toothpaste for no less than two minutes and to refrain from using any other oral care agents until the end of the experiment. Assessment of the indices (OHI-s, API, mSBI) and the collection of material for microbiological examination were performed upon the initial visit (T1), after 7 days (T2) and after 4 weeks (T3) from the initial visit. Every time, patients were examined for regression of lesions, frame of mind and possible side effects, which were recorded on the survey form. Collection of microbiological examination material was performed with a sterile swab equipped with transport medium. The material was collected from mouth floor mucosa with the tongue raised to the palate.

### 4.3. Clinical Examination Protocol

The measurement of given indices was performed with a standard WHO periodontal probe. OHI-S (according to Greene and Vermilion) was determined with Debris Index (DI-s) and Calculus Index (CI-s), by adding the values obtained (DI-s + CI-s). The following six teeth were examined: 16, 11, 26, 36, 31, 46. Criteria for the assessment of DI-s and CI-s for both parameters are as follows: 0—no debris or calculus present, 1—soft debris or supragingival calculus covering not more than one-third of the tooth surface, 2—soft debris or supragingival calculus covering not more than two-thirds of the tooth surface or individual calculus flecks, 3—debris or supragingival calculus covering more than two-thirds of the tooth surface or a heavy band of subgingival calculus.

The API assesses the incidence of dental plaque on occlusal surfaces: in quadrants 1 and 3 on occlusal surfaces on the side of the oral cavity proper and in quadrants 2 and 4 on occlusal surfaces on the side of the vestibule. The following formula is used to calculate the API value (according to Lange): (%) API = the sum of interdental spaces with plaque/the sum of all examined surfaces × 100%. Results obtained were interpreted according to the following scale: API 100–70%—bad oral hygiene. API 70–40%—average hygiene, improvement recommended. API 39–25%—quite good oral hygiene. API <25%—optimal oral hygiene.

Subsequently, bleeding of the interdental papilla was evaluated, based on the mSBI index (by Muhlemann-Son), in quadrants 1 and 3 on occlusal surfaces on the side of the oral cavity proper and in quadrants 2 and 4 on occlusal surfaces on the side of the vestibule. The following scale was used for the calculation: 0—healthy gingiva, no bleeding on probing, 1—healthy looking gingiva, bleeding on probing, 2—change in colour, bleeding on probing, 3—change in colour, slight change in shape, oedema, bleeding on probing, 4—change in colour, obvious change in shape, bleeding on probing, 5—change in colour, marked oedema, bleeding on probing; and the formula used was (%) mSBI = number of bleeding spaces/number of all examined spaces × 100%. Results were interpreted based on the following scale: mSBI 100–50%—general and intensive gingivitis; mSBI 49–30%—general and moderate gingivitis; mSBI 29–10%—localised and mild gingivitis; mSBI <10%—clinically healthy periodontium.

### 4.4. Microbiological Examinations

Microbiological examinations were performed in the Microbiological Laboratory at the Department of Microbiology and Immunology (Zabrze, Poland) with use of classic methods employed in microbiological diagnostics. Material collected from the patients was delivered to the laboratory on the day of collection and immediately cultured on growth media for proliferation and isolation of pure cultures. Aerobic bacteria were proliferated on Columbia Agar solid medium with 5% sheep blood at 37 °C. Anaerobic bacteria were proliferated on Schaedler K3 solid medium with 5% sheep blood at 37 °C in anaerobic conditions obtained with Biomerieux GENbag anaer generators (Marcy l’Étoile, France). Fungi of the *Candida* genus were proliferated and initially identified with use of ChromID Candida chromogenic medium (Biomerieux, Marcy l’Étoile, France). After isolation and proliferation of cultured microorganism strains, their species were identified with use of the following reagent kits: ENTEROtest 24 N, NEFERMtest 24 N, STREPTOtest 24, STAPHYtest 24, ANAEROtest 23, OXItest, PYRAtest and TNW_lite 6.5 computer program for species identification of microorganisms (Erba-Lachema, Brno, Czech Republic). Also, the following Biomerieux (Marcyl’Étoile, France) biochemical tests were used: Katalaza, Slidex Staph Kit, API Candida. Performance and interpretation of results of the tests were carried out according to the manufacturer’s recommendations with diagnostic reagent kits.

### 4.5. Statistical Analysis

The first stage of statistical analysis consisted of verification of the compatibility of the obtained index values and the number of bacteria with normal distribution with use of the Shapiro–Wilk test. Variables with normal distribution were presented with arithmetic mean and standard deviation, whereas nonparametric variables were presented with median and interquartile range. One-way analysis of variance ANOVA and Levene’s test were used to compare the results of the study group with the control group for OHI-s, API and mSBI. The comparison of results between the groups was performed with the Tukey–Kramer method. Results obtained for API, mSBI and OHI-s were compared with Student’s *t*-test for dependent and independent samples. For unrelated variables, the results of the study and control groups were compared with Mann–Whitney U test, Wilcoxon signed-rank test and Friedman’s ANOVA with Kendall’s coefficient of concordance. Observed differences in assessed parameters were deemed to be statistically significant if *p* < 0.05.

## 5. Conclusions

The use of a toothpaste containing ethanolic extract of propolis and tea tree oil helps to maintain oral microbiome balance and eliminate microorganisms causing diseases of the gingiva and hard tooth tissue.

## Figures and Tables

**Table 1:** Comparison of mean API values with Student’s *t*-test for dependent and independent samples in study and control groups.

Oral Hygiene Assessment (Interproximal Spaces) API											
	T1		Mean ± Standard Deviation	T2		Mean ± Standard Deviation	*p* (vs. T1)	T3		Mean ± Standard Deviation	*p* (vs. T1)
Study group (AT preparation)	Optimal	0%	64.58 ± 22.38	Optimal	20%	49.00 ± 25.32	0.006488	Optimal	16%	39.39 ± 20.60	0.000155
	Quite good	12%		Quite good	20%			Quite good	52%		
	Average	48%		Average	44%			Average	24%		
	Bad	40%		Bad	16%			Bad	8%		
Control group (CT preparation)	Optimal	27%	50.72 ± 29.68	Optimal	35%	47.56 ± 29.70	0.572221	Optimal	46%	42.19 ± 28.76	0.181755
	Quite good	19%		Quite good	11%			Quite good	12%		
	Average	27%		Average	31%			Average	23%		
	Bad	27%		Bad	23%			Bad	19%		
**Study vs. Control group (*p*)**	0.066497			0.852609				0.692097			

T1—first examination; T2—second examination after 7 days; T3—third examination after 28 days.

**Table 2:** Comparison of the mSBI values in study and control groups.

Sulcus Bleeding Index Assessment (mSBI)								
	T1		T2		T3		Friedman’s ANOVA Test (*p*)/Kendall’s Coefficient of Concordance	Wilcoxon Signed-Rank Test *p*
Study group (AT preparation)	Normal gingiva SBI <10%	4%	Normal gingiva SBI <10%	8%	Normal gingiva SBI <10%	24%	*p* = 0.00001/0.68367	T1:T2 = 0.017720 T2:T3 = 0.000024 T1:T3 = 0.000014
	Bleeding on probing	96%	Bleeding on probing	92%	Bleeding on probing	76%		
Control group (CT preparation)	Normal gingiva SBI <10%	4%	Normal gingiva SBI <10%	4%	Normal gingiva SBI <10%	8%	*p* = 0.00004/0.38811	T1:T2 = 0.026810 T2:T3 = 0.002238 T1:T3 = 0.002508
	Bleeding on probing	96%	Bleeding on probing	96%	Bleeding on probing	92%		
**Mann–Whitney U test (*p*)**	0.417321		0.049853		0.000002		-	--

T1—first examination; T2—second examination after 7 days; T3—third examination after 28 days.

**Table 3:** Comparison of the OHI-s values in study and control groups.

Oral Hygiene Assessment (Interproximal Spaces) (OHI-s)											
	T1		Mean ± Standard Deviation	T2		Mean ± Standard Deviation	*p* (vs. T1)	T3		Mean ± Standard Deviation	*p* (vs. T1)
Study group (AT preparation)	0–0.5	12%	1.48 ± 0.70	0–0.5	48%	0.66 ± 0.49	0.000001	0–0.5	96%	0.12 ± 0.18	0.000001
	0.6–1	16%		0.6–1	20%			0.6–1	4%		
	1.1–2	44%		1.1–2	32%			1.1–2	0%		
	2.1–3	28%		2.1–3	0%			2.1–3	0%		
Control group (CT preparation)	0–0.5	27%	1.18 ± 0.61	0–0.5	30%	1.09 ± 0.60	0.461274	0–0.5	38%	0.99 ± 0.55	0.186593
	0.6–1	8%		0.6–1	16%			0.6–1	8%		
	1.1–2	57%		1.1–2	46%			1.1–2	54%		
	2.1–3	8%		2.1–3	8%			2.1–3	0%		
**Study vs. Control (*p*)**	0.101110			0.007670				0.000001			

T1—first examination; T2—second examination after 7 days; T3—third examination after 28 days.

**Table 4:** Analysis of homogeneity of variance for OHI-s.

	Analysis of Variance (F)	*p*	Levene’s Test for Homogeneity of Variance	*p*
**OHI-s (T1)**	2.79204	0.101110	0.15039	0.699845
**OHI-s (T2)**	7.73369	0.007670	1.85717	0.179182
**OHI-s (T3)**	56.25920	0.000001	64.33246	0.000001

**Table 5:** Microorganisms found in the samples taken from mouth floor mucosa of patients from study and control groups.

Microorganisms	Study Group (AT Preparation)				Control Group (CT Preparation)			
	T1	T2	T3	Together	T1	T2	T3	Together
**Gram (+) cocci**								
*Gemella* sp.	1	1	1	**3**	1	0	0	**1**
*Leuconostoc* sp.	1	0	0	**1**	0	0	0	**0**
*Staphylococcus aureus MSSA*	2	1	2	**5**	2	4	2	**8**
*Staphylococcus epidermidis MSCNS*	1	0	0	**1**	0	1	0	**1**
*Streptococcus acidominimus*	1	1	1	**3**	0	0	1	**1**
*Streptococcus mitis*	17	15	15	**47**	9	12	16	**37**
*Streptococcus oralis*	2	2	1	**5**	1	3	0	**4**
*Streptococcus sanguinis*	10	5	7	**22**	4	6	7	**17**
*Streptococcus salivarius*	6	8	5	**19**	10	10	6	**26**
*Peptococcus niger*	0	0	1	**1**	0	1	0	**1**
*Streptococcus vestibularis*	0	0	0	**0**	1	1	0	**2**
**Gram (−) cocci**								
*Moraxella catarrhalis*	0	0	0	**0**	0	0	1	**1**
*Neisseria subflava*	16	17	22	**55**	24	26	26	**76**
*Neisseria sicca*	1	1	1	**3**	0	0	0	**0**
*Neisseria mucosa*	1	0	0	**1**	0	0	0	**0**
*Neisseria parvula*	0	0	1	**1**	0	0	0	**0**
*Veillonella parvula*	1	1	2	**4**	3	4	0	**7**
**Gram (+) bacilli and rods**								
*Actinomyces naeslundii*	8	8	6	**22**	8	9	17	**34**
*Actinomyces israelii*	2	3	2	**7**	3	0	7	**10**
*Atopobium minutum*	0	0	1	**1**	1	0	0	**1**
*Actinomyces odontolyticus*	0	1	0	**1**	0	0	1	**1**
*Actinomyces viscosus*	1	0	0	**1**	0	0	1	**1**
*Atopobium parvulum*	1	1	0	**2**	1	0	0	**1**
*Bifidobacterium breve*	7	5	1	**13**	2	1	3	**6**
*Bifidobacterium longum*	2	0	0	**2**	1	1	3	**5**
*Bifidobacterium dentium*	3	3	3	**9**	2	1	1	**4**
*Bifidobacterium odolescentis*	1	0	0	**1**	0	3	1	**4**
*Blautia producta*	1	0	1	**2**	2	0	1	**3**
*Clostridium perfringens*	5	2	1	**8**	1	6	4	**11**
*Clostridium butyricum*	0	0	0	**0**	0	1	1	**2**
*Clostridium chauvoei*	0	0	1	**1**	0	0	1	**1**
*Clostridium ramosum*	0	0	0	**0**	0	0	1	**1**
*Clostridium tertium*	1	0	0	**1**	0	0	0	**0**
*Clostridium sporogenes*	0	1	1	**2**	0	0	0	**0**
*Clostridium difficile*	0	0	0	**0**	0	0	1	**1**
*Leptotrichia buccalis*	0	1	0	**1**	0	0	0	**0**
*Lactobacillus acidophilus*	4	2	3	**9**	6	2	0	**8**
*Lactobacillus fermentum*	0	0	1	**1**	0	1	1	**2**
*Lactobacillus catenaformis*	0	0	1	**1**	0	0	0	**0**
*Propionibacterium propionicum*	0	1	1	**2**	1	0	2	**3**
*Propionibacterium acnes*	0	2	0	**2**	0	1	1	**2**
*Propionibacterium granulosum*	0	0	1	**1**	0	1	0	**1**
*Pseudoflavonifractor capillosus*	1	0	0	**1**	0	2	0	**2**
**Gram (−) bacilli and rods**								
*Bacteroides uniformis*	0	0	0	**0**	1	0	0	**1**
*Bacteroides ovatus*	0	3	1	**4**	0	1	0	**1**
*Capnocytophaga ochracea*	2	0	1	**3**	3	2	1	**6**
*Campylobacter ureolyticus*	0	0	0	**0**	2	0	1	**3**
*Campylobacter gracilis*	3	3	0	**6**	1	4	3	**8**
*Escherichia coli*	1	2	0	**3**	0	1	0	**1**
*Eikenella corrodens*	0	1	0	**1**	0	0	0	**0**
*Klebsiella oxytoca*	0	1	0	**1**	0	0	0	**0**
*Mitsuokella multacida*	2	4	5	**11**	1	1	3	**5**
*Prevotella oralis*	1	1	2	**4**	2	1	0	**3**
*Prevotella melaninogenica*	0	0	2	**2**	0	1	0	**1**
*Parabacteroides distasonis*	2	0	1	**3**	0	0	0	**0**
*Pseudomonas putida*	0	0	1	**1**	0	0	0	**0**
**Fungi**								
*Candida albicans*	3	1	0	**4**	2	2	2	**6**
**Number of isolated strains:**	111	98	96	**305**	95	110	116	**321**
